# Association of IL‐33 in modeling type‐2 airway inflammation and pulmonary emphysema in mice

**DOI:** 10.1002/iid3.1252

**Published:** 2024-04-23

**Authors:** Chika Miyaoka, Masato Watanabe, Keitaro Nakamoto, Yuki Yoshida, Aya Hirata, Jumpei Aso, Hiroki Nunokawa, Manabu Ishida, Koujiro Honda, Saori Takata, Takeshi Saraya, Haruyuki Ishii

**Affiliations:** ^1^ Department of Respiratory Medicine Kyorin University Faculty of Medicine Mitaka City Tokyo Japan

**Keywords:** asthma, COPD, IFNγ, IL‐13, IL‐33, IL‐4, IL‐5, innate immunity

## Abstract

We developed pulmonary emphysema and a type 2 airway inflammation overlap mouse model. The bronchoalveolar lavage (BAL) interleukin 13 (IL‐13), IL‐4, and IL‐5 levels in the overlap model were higher than in the pulmonary emphysema model and lower than in the type 2 airway inflammation model, but IL‐33 level in the lung was higher than in other models. IL‐33 and interferon‐γ (IFNγ) in lungs may control the severity of a type 2 airway inflammation in lung.

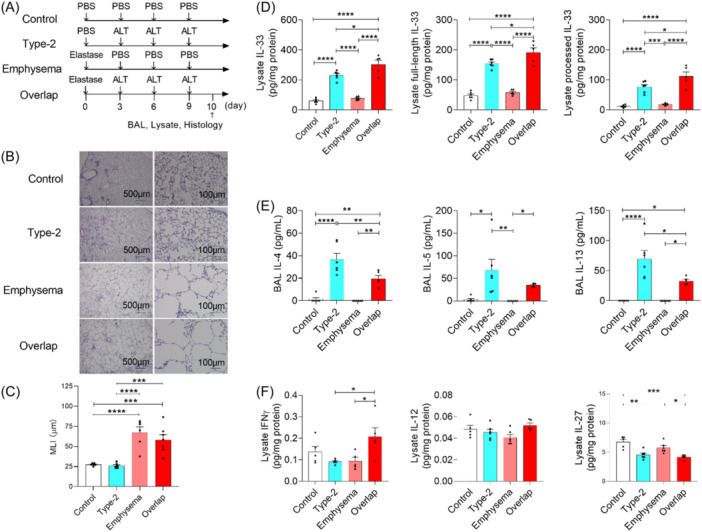

To the Editor,

Asthma and chronic obstructive pulmonary disease (COPD) are common chronic airway diseases that frequently coexist, and together they are referred to specifically as asthma–COPD overlap (ACO). Because asthma and COPD are heterogeneous diseases, ACO pathogenesis is more complex and its immune mechanisms and optimal treatments remain largely unknown. Interleukin 33 (IL‐33) is an epithelium‐derived pro‐type 2 cytokine that functions as an alarmin. We previously reported that the sputum of patients with COPD or ACO contains high levels of IL‐33.^1^ *Alternaria alternata*, an airborne allergen, triggers IL‐33‐induced asthma‐like type 2 airway inflammation by activating group 2 innate lymphocyte (ILC2).^2^ We hypothesized that IL‐33‐derived inflammation is involved in ACO pathogenesis. We established a type‐2 airway inflammation and pulmonary emphysema overlap model by combining *Alternaria*‐derived type 2 innate airway inflammation and porcine pancreatic elastase (PPE)‐induced pulmonary emphysema to test this hypothesis. We also cultured normal human bronchial epithelial (NHBE) cells to assess IL‐33 expression and release after exposure to neutrophil elastase (NE).

We first confirmed that the emphysema model developed pulmonary emphysema (Supporting Information S1: Figure S1A–C). IL‐33 was upregulated in the lungs from Days 1 to 3 (Supporting Information S1: Figure S1D). Most of the IL‐33 was intracellular full‐length and the remainder was extracellularly processed (Supporting Information S1: Figure S1E, S1F). We also assessed immune cell counts and cytokine levels in the bronchoalveolar lavage (BAL) fluid and lung lysate (Supporting Information S1: Figure S2A, S2B). In culture experiments, human bronchial epithelial cells (Table E1) exhibited stable intracellular IL‐33 expression (Supporting Information S1: Figure S1G). NE promoted both the expression and release of IL‐33 (Supporting Information S1: Figure S1H). These findings indicate that NE upregulate intracellular IL‐33 in the lungs, and this is partially released in the airspaces.

We next hypothesized that the emphysema model would release more IL‐33 in the alveoli after *Alternaria alternata* inhalation. We compared inflammation patterns in the type‐2 airway inflammation (*A. alternata*), emphysema (PPE), and overlap models (both) (Figure 1A and Supporting Information S1: Figures S3A, S3B). The emphysema and overlap models developed pulmonary emphysema (Figure 1B, C). Lung IL‐33 concentrations were higher in the emphysema model than in the control model from hour 1 to 7 (total IL‐33, Days 1–3; full‐length IL‐33, Days 3–7; processed IL‐33, Hour 1 to Day 3, Supporting Information S1: Figure S1D–F), but this was no longer the case on day 10 (Figure 1D). Lung IL‐33 levels (total, full‐length, and processed) were higher in the overlap model than in the others (Figure 1D). Paradoxically, the overlap model showed lower IL‐4, IL‐5, and IL‐13 levels in the BAL fluid than the type‐2 airway inflammation model (Figure 1E). We then suspected that ILC2‐antagonizing cytokines^2^ were upregulated in the overlap model lung. As expected, interferon‐γ (IFNγ), but not IL‐12 or IL‐27, was upregulated in the overlap model lung (Figure 1F). This indicates that IL‐33 and IFNγ may positively and negatively regulate, respectively, type 2 airway inflammation in our overlap model.

**Figure 1:**
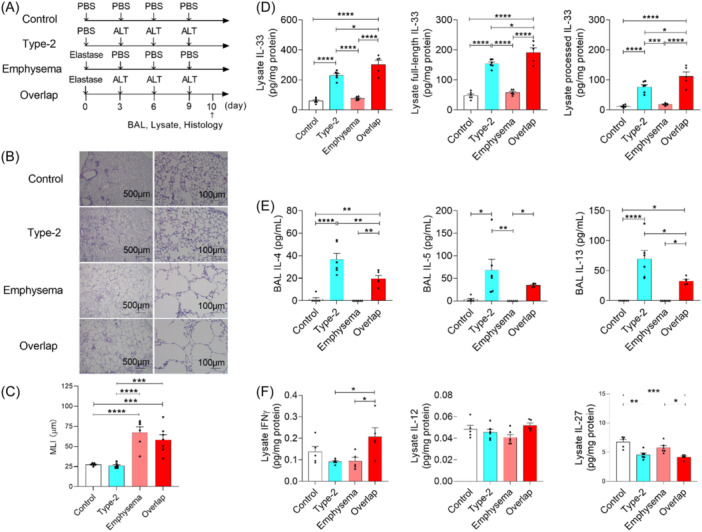
Airway inflammation in mouse models of asthma, emphysema, and asthma–chronic obstructive pulmonary disease overlap (ACO). (A) Protocol for assessing type‐2 airway inflammation (Type‐2), emphysema, and overlap mouse models. (B, C) Pathologies (B) and mean linear intercept (MLI, C) of the mouse lungs (*n* = 6–8 mice). Bars, 500 μm (×40) and 100 μm (×200). (D) Interleukin 33 (IL‐33) levels in lung lysate. Total, full‐length, and processed IL‐33 (left, middle, and right, respectively) were calculated as described in Figure 1. (E) IL‐4, IL‐5, and IL‐13 levels in bronchoalveolar lavage (BAL) fluid (*n* = 5–6 mice). (F) Interferon γ (IFNγ), IL‐12, and IL‐27 levels in lung lysate (*n* = 5–6 mice). Data are pooled from three experiments and expressed as mean ± standard error of the mean (C–F). ****p < .0001, ***p < .001, **p < .01, *p < .05. *p* values were calculated using one‐way analysis of variance and post hoc Tukey tests (C–F). ALT, *Alternaria alternata*; PBS, phosphate‐buffered saline; Type‐2, type‐2 airway inflammation; Overlap, type‐2 airway inflammation and pulmonary emphysema overlap.

A previous study reported that the papain‐induced ACO model develops allergic asthma.^3^ Another study demonstrated that the PPE and ovalbumin‐induced ACO model also develops allergic asthma.^4^ Our overlap model used the previously reported *A. alternata* administration schedule.^2^ This administration schedule increases the number of ILC2 and CD4^+^ T cells containing IL‐5 and ‐13 intracellularly in BAL fluid.^2^ Thus, our ACO model may have innate and allergic asthma aspects.

More importantly, our overlap model showed that type 2 airway inflammation is stronger than in the emphysema model and weaker than in the type‐2 airway inflammation model. These results are consistent with clinical observations that ACO patients exhibit higher fractional exhaled nitric oxide (FeNO) levels than COPD patients and lower FeNO levels than asthmatics^5^, ^6^, ^7^, because FeNO is a biomarker of type‐2 inflammation^8^ and IL‐13 derives inducible nitric oxide synthase production in human bronchial epithelial cells.^9^ Our overlap model had upregulated IL‐33 and IFNγ levels in the lungs, and these positively and negatively regulate ILC2, respectively.^2^ These results suggest that IL‐33 and IFNγ may mediate FeNO levels in ACO patients.

Our overlap and innate type‐2 airway inflammation models exhibited upregulated IL‐33 release in the alveoli. Monoclonal anti‐IL‐33 antibodies reduce exacerbation in patients with moderate‐to‐severe COPD^10^ or moderate‐to‐severe asthma.^11^ Thus, IL‐33‐targeting therapies may reduce exacerbation in patients with ACO (i.e., COPD plus innate asthma), although further investigations are required.

This study was subject to some limitations. PPE does not trigger all the physiological events associated with cigarette smoke.^12^ Thus, our PPE‐induced emphysema model was not fully relevant to human COPD. We did not assess airway hypersensitivity in our mouse model after *A. alternata* administration. Thus, this was not an asthma model but an asthma‐like type 2 airway inflammation model.

In conclusion, our overlap model shows airway inflammation relevant to clinical ACO, and we found that IL‐33 is a possible therapeutic target in patients with ACO.

## AUTHOR CONTRIBUTIONS

**Chika Miyaoka**: Conceptualization (equal); data curation (equal); formal analysis (equal); investigation (equal); resources (equal); validation (equal); visualization (equal); writing—original draft preparation (equal). **Masato Watanabe**: Conceptualization (equal); data curation (supporting); funding acquisition (lead); investigation (equal); methodology (lead); project administration (lead); supervision (lead); writing—original draft preparation (equal); writing—review & editing (lead). **Keitaro Nakamoto**: Conceptualization (supporting); formal analysis (equal); resources (equal); validation (equal). **Yuki Yoshida**: Data curation (equal); formal analysis (equal); resources (supporting). **Aya Hirata**: Methodology (supporting); resources (supporting). **Jumpei Aso**: Data curation (equal); validation (supporting). **Hiroki Nunokawa**: Validation (supporting). **Manabu Ishida**: Formal analysis (supporting); investigation (supporting). **Kojiro Honda**: Formal analysis (supporting). **Saori Takata**: Investigation (supporting). **Takeshi Saraya**: Investigation (supporting); writing—review & editing (supporting). **Haruyuki Ishii**: Writing—review & editing (supporting).

## CONFLICT OF INTEREST STATEMENT

The authors declare no conflict of interest.

## ETHICS STATEMENT

The Experimental Animal Ethics Committee of Kyorin University (No. 236).

## Supporting information

Supporting information.

## Data Availability

The data supporting this study's findings from the corresponding author upon reasonable request.

## References

[1] Watanabe M , Nakamoto K , Inui T , et al. Soluble ST2 enhances IL‐33‐induced neutrophilic and pro‐type 2 inflammation in the lungs. Allergy. 2022;77:3137‐3141. 35661175 10.1111/all.15401 PMC9796337

[2] Moro K , Kabata H , Tanabe M , et al. Interferon and IL‐27 antagonize the function of group 2 innate lymphoid cells and type 2 innate immune responses. Nature Immunol. 2016;17:76‐86. 26595888 10.1038/ni.3309

[3] Fukuda K , Matsuzaki H , Mikami Y , et al. A mouse model of asthma‐chronic obstructive pulmonary disease overlap induced by intratracheal papain. Allergy. 2021;76:390‐394. 32740929 10.1111/all.14528

[4] Jo YS , Rhee CK , Yoon HK , et al. Evaluation of asthma‐chronic obstructive pulmonary disease overlap using a mouse model of pulmonary disease. J Inflamm. 2022;19:25. 10.1186/s12950-022-00322-x PMC9728005 36474247

[5] Chen FJ , Huang XY , Liu YL , Lin GP , Xie CM . Importance of fractional exhaled nitric oxide in the differentiation of asthma‐COPD overlap syndrome, asthma, and COPD. Int J Chron Obstruct Pulmon Dis. 2016;11:2385‐2390. 27713629 10.2147/COPD.S115378 PMC5045026

[6] Duong‐Quy S , Tran Van H , Vo Thi Kim A , Pham Huy Q , Craig TJ . Clinical and functional characteristics of subjects with asthma, COPD, and asthma‐COPD overlap: a multicentre study in Vietnam. Can Respir J. 2018;2018:1‐11. 10.1155/2018/1732946 PMC5901814 29808101

[7] de Llano LP , Cosío BG , Iglesias A , et al. Mixed Th2 and non‐Th2 inflammatory pattern in the asthma‐COPD overlap: a network approach. Int J Chron Obstruct Pulmon Dis. 2018;13:591‐601. 29483774 10.2147/COPD.S153694 PMC5813946

[8] Chung KF . Increasing utility of FeNO as a biomarker of type‐2 inflammation in severe asthma. Lancet Respir Med. 2021;9:1083‐1084. 34181878 10.1016/S2213-2600(21)00170-3

[9] Chibana K , Trudeau JB , Mustovitch AT , et al. IL‐13 induced increases in nitrite levels are primarily driven by increases in inducible nitric oxide synthase as compared with effects on arginases in human primary bronchial epithelial cells. Clin Exp Allergy. 2008;38:936‐946. 18384429 10.1111/j.1365-2222.2008.02969.x PMC11934259

[10] Rabe KF , Celli BR , Wechsler ME , et al. Safety and efficacy of itepekimab in patients with moderate‐to‐severe COPD: a genetic association study and randomised, double‐blind, phase 2a trial. Lancet Respir Med. 2021;9:1288‐1298. 34302758 10.1016/S2213-2600(21)00167-3

[11] Wechsler ME , Ruddy MK , Pavord ID , et al. Efficacy and safety of itepekimab in patients with moderate‐to‐severe asthma. N Engl J Med. 2021;385:1656‐1668. 34706171 10.1056/NEJMoa2024257

[12] Wright JL , Cosio M , Churg A . Animal models of chronic obstructive pulmonary disease. Am J Physiol Lung Cell Mol Physiol. 2008;295:L1‐L15. 18456796 10.1152/ajplung.90200.2008 PMC2494776

