# Supplementary material for: Association of IL‐33 in modeling type‐2 airway inflammation and pulmonary emphysema in mice
Source: Immun Inflamm Dis. 2024 Apr 23;12(4):e1252. doi: 10.1002/iid3.1252 (PMC11037248; doi:10.1002/iid3.1252)
Supplement: Supplementary file 1 — Supporting information. [file IID3-12-e1252-s001.docx]

**Online Supplement**

**Title:** Association of IL-33 in modeling type-2 airway inflammation and pulmonary emphysema in mice

## Authors:

Chika Miyaoka, MD^1^, Masato Watanabe, MD, PhD^1^, Keitaro Nakamoto, MD, PhD^1^, Yuki Yoshida, MD^1^, Aya Hirata MD, PhD^1^, Jumpei Aso, MD, PhD^1^, Hiroki Nunokawa, MD, PhD^1^, Manabu Ishida, MD^1^, Koujiro Honda, MD, PhD^1^, Saori Takata, MD^1^, Takeshi Saraya, MD, PhD^1^, Haruyuki Ishii, MD, PhD^1^

## Institution:

1. Department of Respiratory Medicine, Kyorin University Faculty of Medicine, 6-20-2 Sinkawa, Mitaka City, Tokyo, 181-8611, Japan

# Methods

## Mice

Seven- to eight-week-old male C57BL/6J (wild-type) mice were obtained from CLEA Japan. All animal experiments were approved by the Experimental Animal Ethics Committee of Kyorin University (No. 236). The protocol was prepared before the study, and this protocol was registered in the Experimental Animal Ethics Committee of Kyorin University. Mouse experiments were conducted as previously described, with some exceptions as follows ([1](#_ENREF_1))**.** Acclimatisation periods to experimental locations are one week. The mice were intratracheally administered with three units of porcine pancreatic elastase (PPE; Elastin Products Company, Owensville, MO, USA) in phosphate-buffered saline (PBS, 50 μl) or PBS only. After the treatments, mice in the cage were in the breeding room at room temperature. After 1 h and 1, 3, and 7 d, the mouse lungs were lavaged twice with 1.0 ml saline and the bronchoalveolar lavage (BAL) fluid was collected. A total of 100 mice (10-12 mice per group) were used for this experiment. Red cells in the BAL fluid were lysed with 100 μl red blood cell lysis buffer (BioLegend, San Diego, CA, USA) for 1 min, after which 900 μl PBS was added. This process was conducted a total of six (day 1) or three times (1 h, days 3 and 7). Cells in the BAL fluid were counted using an automated cell counter (Bio-Rad Laboratories, Hercules, CA, USA), and the differential leukocyte count was determined from smear slides stained with Diff-Quik solution (Sysmex, Kobe, Japan). The right lungs were homogenized in 0.9 ml of T-PER Tissue Protein Extraction Reagent (Thermo Fisher Scientific, Waltham, MA, USA) to measure cytokines and proteins. The left lungs were stored at −80 °C for further analysis. After 21 d, the trachea was cannulated and the lungs were infused with PBS containing 4% paraformaldehyde (fixation buffer) at 43 cm of water pressure. A total of 16 mice (8 mice per group) were used for this experiment. The heart, lungs, and trachea were removed *en bloc* and stored in fixation buffer for 48 h. The left lung was embedded in paraffin and 4 μm-thick sections were cut and stained with hematoxylin and eosin (H&E). In another experiment, the mice were intratracheally administered with PPE (3 units/PBS 50 μl) or PBS (day 0), after which 80 μg/50 μl *Alternaria alternata* extract (ITEA, Tokyo, Japan) or PBS was administered on days 3, 6, and 9. On day 10, the BAL fluid and lung lysate were obtained as described above. A total of 23 mice (5-6 mice per group) were used for this experiment. Red cell lysis was performed using 400 μl red cell lysis buffer and 4 ml PBS twice. Lung pathological specimens were obtained as described above by conducting the same experiments without obtaining the BAL fluid or lung lysate. A total of 26 mice (6-8 mice per group) were used for this experiment. All experiments were repeated at least twice, and the pooled data were analyzed. No inclusion and exclusion criteria for mice were used. Dead mice were excluded during each experiment. Age- and sex-matched mice were randomly assigned to each experiment group. Sample sizes were determined based on previous literature with similar experiments ([2](#_ENREF_2), [3](#_ENREF_3)).

## Mean linear intercept

The H&E-stained slides were digitally scanned under an all-in-one fluorescence microscope (KEYENCE, Osaka, Japan). Ten fields of view (×200 magnification; 547.5 × 730 μm) not including blood vessels and bronchi (> 150 μm) were randomly selected for each slide. The mean linear intercept (MLI) for each field of view was objectively calculated using a previously described semiautomated protocol ([4](#_ENREF_4)) and ImageJ software (<https://imagej.nih.gov/ij/download.html>). Briefly, 19 vertical and 14 horizontal lines were overlaid on one field of view, and the number of intercepts on these lines were counted automatically. We repeated this process for 10 fields of view (i.e., a total of 330 lines) for each slide and calculated the MLI for that slide by dividing the total length of the lines by the total number of intercepts.

## High-sensitivity ELISA

Mouse CXCL1, CCL2, CCL20, IFNγ, and IL-33, -4, -5, -12, -13, and -27 were quantified via high-sensitivity ELISA as previously described ([1](#_ENREF_1)).

## Protein assay

Protein concentration in the lung lysate was determined using a protein assay dye reagent concentrate (Bio-Rad, Hercules, CA, USA).

**Cytotoxicity assay**

Cytotoxicity was assessed using [4,5-dimethylthiazol-2-yl]-2,5-diphenyltetrazolium bromide (MTT) (Sigma, St. Louis, MO) or LDH-Glo™ Cytotoxicity Assay (Promega, Madison, WI) as manufacture’s recommendation.

## Western blot

IL-33 was detected using anti-mouse IL-33 (AF3626) or anti-human IL-33 (MAB36253) antibodies and horseradish peroxidase (HRP)-conjugated anti-goat IgG antibody (all from R&D Systems). Mouse and human β-actins were detected using HRP-conjugated rabbit anti-β-actin antibody (Cell Signaling Technology, Danvers, MA, USA). Full-length (37 kDa) and processed (18 kDa) IL-33 were confirmed by referring to the BAL fluid containing only processed IL-33, which was obtained from the mice 1 h after intratracheal administration of *A. alternata* (Fig S1E). The BAL fluid was concentrated using an ultrafiltration column and subjected to immunoblotting.

## Cell culture

Normal human bronchial epithelial (NHBE) cells (CC-2540; Lonza, Basel, Switzerland) were cultured in 24- or 6-well plates (Iwaki, Shizuoka, Japan) using the Bronchial Epithelial Cell Growth Medium BulletKit (BEGM, Lonza) as per the supplier’s recommendations. After reaching 60–90% confluence, the NHBE cells were medium-exchanged into corticosteroid-free BEGM for 24 h. These cells were stimulated with neutrophil elastase (NE) from purulent human sputum (Elastin Products Company, Owensville, MO, USA) for 2 h. To detect IL-33 in the lysate via western blotting, the NHBE cells were cultured for 3 d with BEGM. The cell lysate was collected using radioimmunoprecipitation assay buffer (Wako).

Table E1. Normal human bronchial epithelial cell donors

| Smoking | Age | Sex | HLA-A/B/C | Diabetes | Heart Disease | Hypertension | Race | Alcohol | Smoke |
| --- | --- | --- | --- | --- | --- | --- | --- | --- | --- |
| Smoker | 79 | F | Unknown | N | N | N | C | Y | Y |
| Smoker | 70 | M | A01, A03; B07, B08; DR11, DR15 | Y | Unknown | Unknown | C | Y | Y |
| Smoker | 60 | F | A03, A23; B42, B45; C07, C17 | N | Y | Y | B | Y | Y |
| Smoker | 66 | M | Unknown | N | Unknown | Unknown | C | Y | Y |

## Statistics

Numerical data were evaluated for normality and variance by using Shapiro–Wilk and Kolmogorov–Smirnov tests and are presented as mean ± standard error of the mean (SEM; parametric data). Statistical comparisons were made using Student’s *t*-test, one-way analysis of variance (ANOVA) with the post hoc Tukey test or post hoc Holm–Sidak tests, or two-way ANOVA with the post hoc Dunnett test. P-values of < 0.05 were considered statistically significant. Data were analyzed by conducting appropriate tests using GraphPad Prism v. 8.4.3 (GraphPad Software, San Diego, CA, USA).

**Figure legends**


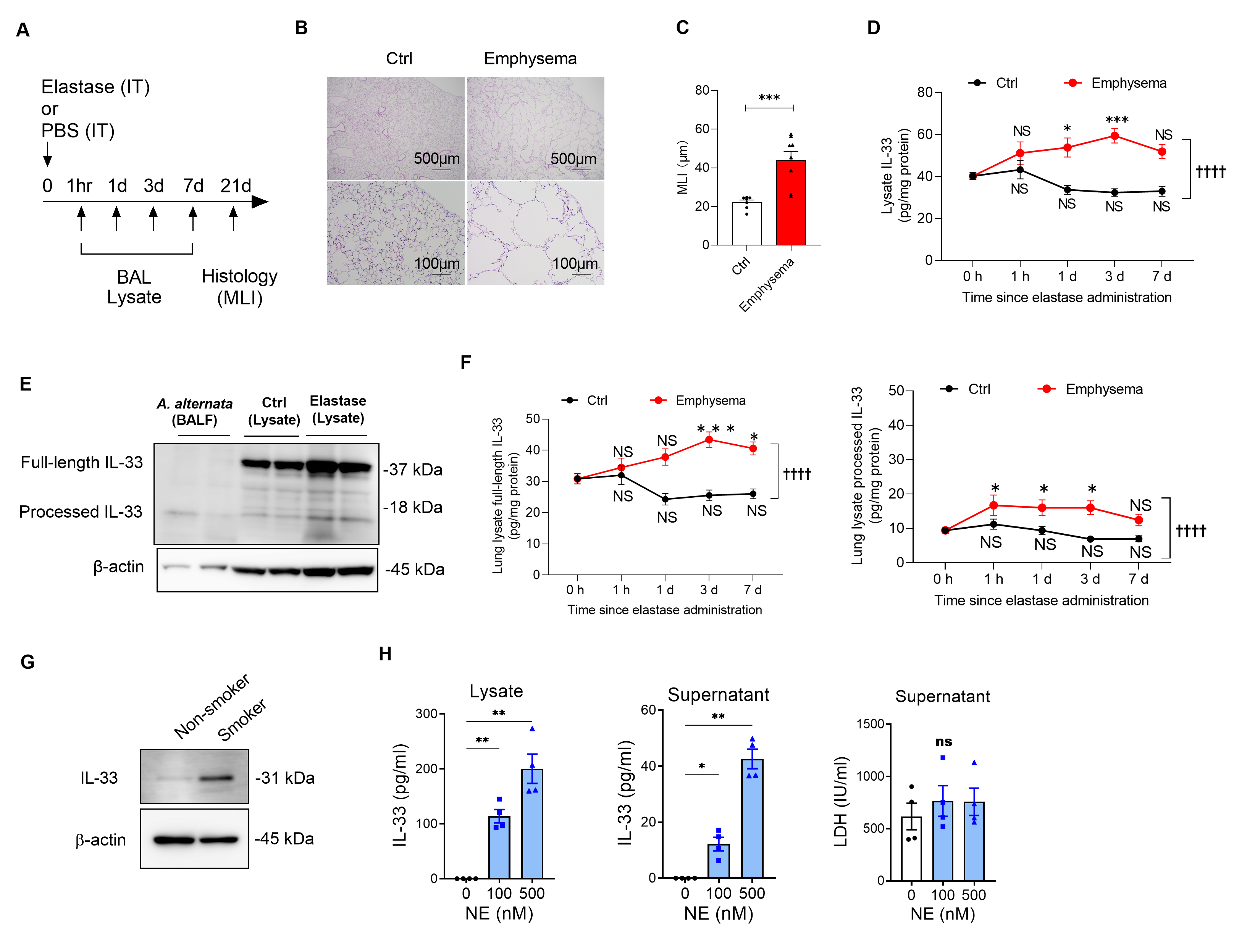


**Figure S1. Elastase augment intracellular expression and extracellular release of IL-33 in the lungs.**

(A) Protocol for assessing the elastase-induced pulmonary emphysema model. (B, C) Pathologies (B) and mean linear intercept (MLI, C) of the emphysema (elastase) model or control (Ctrl, phosphate-buffered saline) mice lungs (*n* = 7–8 mice). Bars, 500 μm (×40) and 100 μm (×200). (D) Standardized IL-33 levels in the lung lysate (*n* = 10–12 mice). (E) Western blotting detected full-length IL-33 (37 kDa), processed IL-33 (18 kDa), and β-actin (45 kDa) in the lung lysate of the emphysema (elastase) model or control (Ctrl, phosphate-buffered saline) mice (day 3) and BAL fluid of mice after intratracheal administration of *A. alternata* (1 h). (F) Standardized full-length (left) and processed IL-33 (right) levels in the lung lysate (*n* = 10–12 mice). Levels of full-length and processed IL-33 on the immunoblot were measured via densitometry, and their ratios were multiplied by the standardized IL-33 levels in the lungs. (G) Immunoblot detection of IL-33 and β-actin in normal human bronchial epithelial (NHBE) cells from two donors (nonsmoker and smoker). (H) IL-33 levels in NHBE cell lysate and culture supernatant. The cells were incubated with neutrophil elastase (NE) for 2 h (*n* = 4 wells/group). Data are pooled from two experiments (C, D, and F) or four experiments using different donors (H) and are expressed as mean ± standard error of the mean (C, D, F and H). **** p < 0.0001, *** p < 0.001, ** p < 0.01, * p < 0.05 (F, vs. 0 h; H, vs. zero control). ^††††^ p < 0.0001 (vs. phosphate-buffered saline [PBS]). P-values were calculated using Student’s *t*-test (C), two-way analysis of variance (ANOVA) and post hoc Dunnett tests (D, F), or one-way ANOVA and post hoc Holm–Sidak tests (H). PBS, phosphate-buffered saline.

**
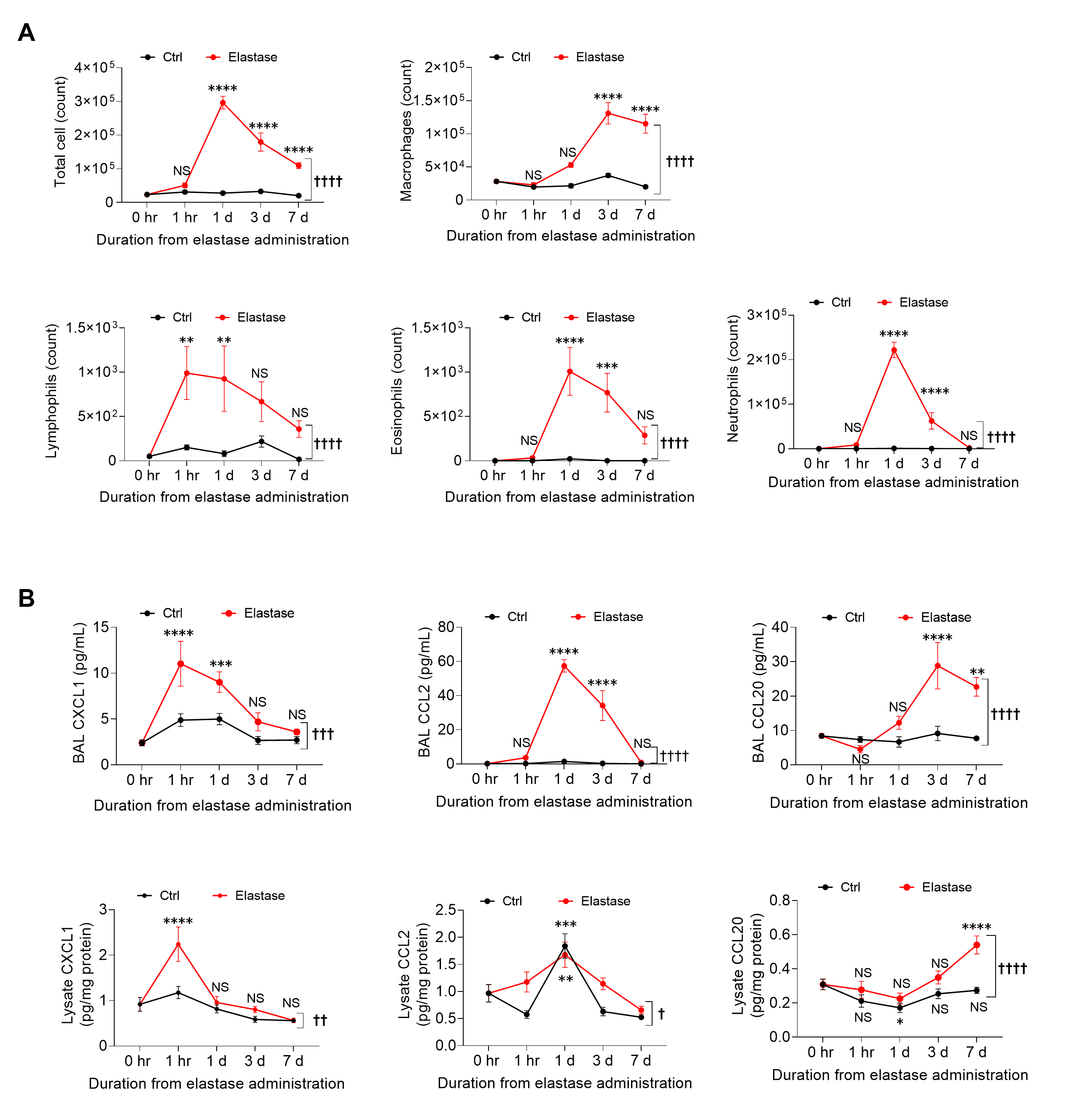
**

**Figure S2. Immune cell counts and cytokine levels in bronchoalveolar lavage (BAL) fluid from the emphysema model.** (A, B) Immune cells (A) or cytokines (B) in BAL fluid of emphysema model or control mice. Data are pooled from two experiments and are expressed as mean ± standard error of the mean (A, B). **** p < 0.0001, *** p < 0.001, ** p < 0.01, * p < 0.05 (vs. 0 h). ^††††^ p < 0.0001 (vs. PBS). P-values were calculated using two-way analysis of variance and post hoc Dunnett tests.


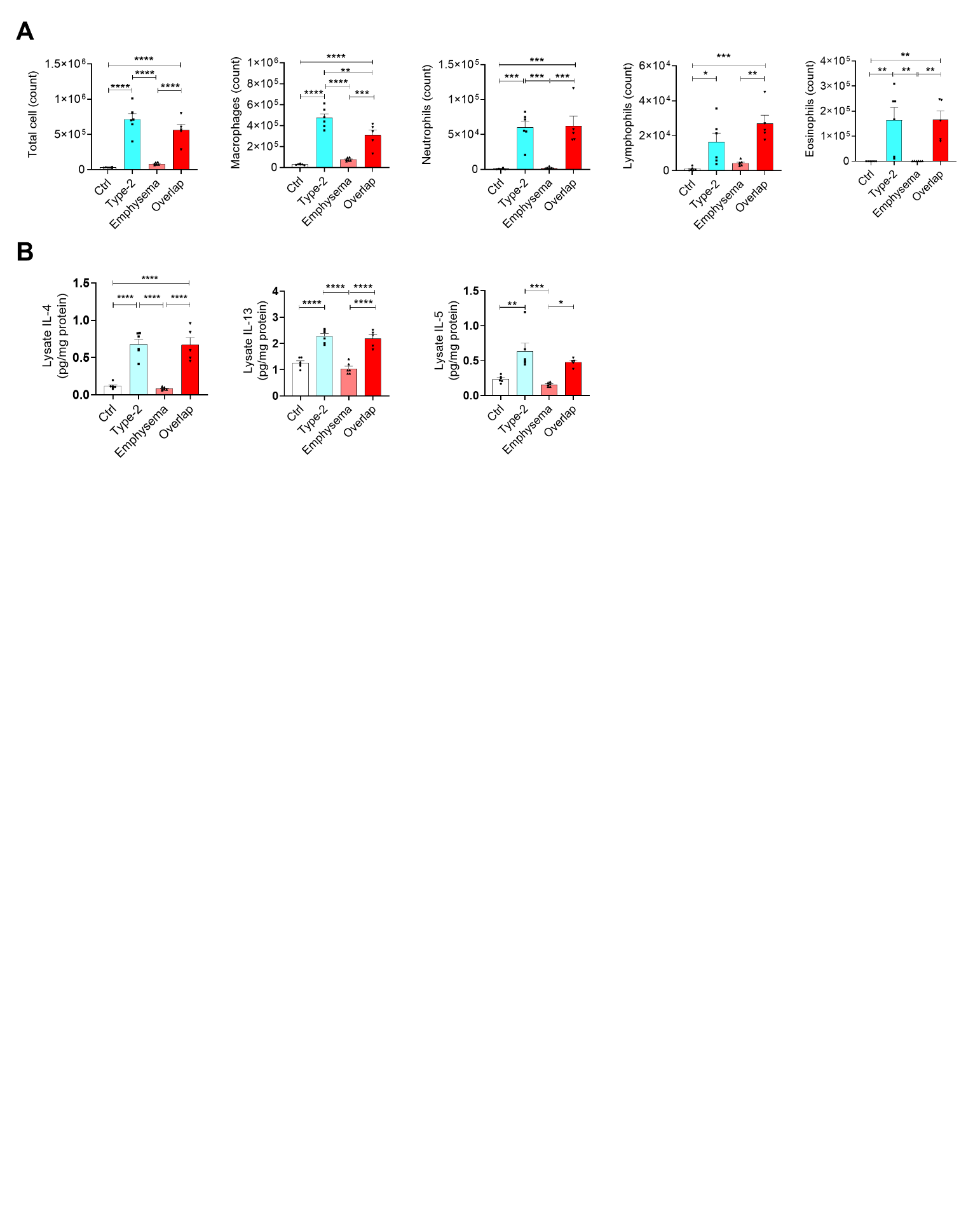


**Figure S3. Immune cell counts in bronchoalveolar lavage (BAL) fluid and cytokine levels in the lungs of type-2 airway inflammation, emphysema, and overlap models.**

(A–B) Immune cell counts in BAL fluid (A) and cytokine levels in the lung lysates (B) of type-2 airway inflammation, emphysema, and overlap models. Data are pooled from three experiments and are expressed as mean ± standard error of the mean. **** p < 0.0001, *** p < 0.001, ** p < 0.01, * p < 0.05. P-values were calculated using one-way analysis of variance and post hoc Tukey tests. Ctrl, control, phosphate-buffered saline; PBS, phosphate-buffered saline. Type-2, type-2 airway inflammation; Overlap, type-2 airway inflammation–pulmonary emphysema overlap.

# Reference

1. Watanabe M, Nakamoto K, Inui T, Sada M, Chibana K, Miyaoka C, Yoshida Y, Aso J, Nunokawa H, Honda K, Nakamura M, Tamura M, Hirata A, Oda M, Takata S, Saraya T, Kurai D, Ishii H, Takizawa H. Soluble ST2 enhances IL-33-induced neutrophilic and pro-type 2 inflammation in the lungs. *Allergy* 2022; 77: 3137-3141.

2. Tamura K, Matsumoto K, Fukuyama S, Kan OK, Ishii Y, Tonai K, Tatsuta M, Enokizu A, Inoue H, Nakanishi Y. Frequency-dependent airway hyperresponsiveness in a mouse model of emphysema and allergic inflammation. *Physiol Rep* 2018; 6.

3. Tu X, Kim RY, Brown AC, de Jong E, Jones-Freeman B, Ali MK, Gomez HM, Budden KF, Starkey MR, Cameron GJM, Loering S, Nguyen DH, Nair PM, Haw TJ, Alemao CA, Faiz A, Tay HL, Wark PAB, Knight DA, Foster PS, Bosco A, Horvat JC, Hansbro PM, Donovan C. Airway and parenchymal transcriptomics in a novel model of asthma and COPD overlap. *J Allergy Clin Immunol* 2022; 150: 817-829.e816.

4. Crowley G, Kwon S, Caraher EJ, Haider SH, Lam R, Batra P, Melles D, Liu M, Nolan A. Quantitative lung morphology: semi-automated measurement of mean linear intercept. *BMC Pulm Med* 2019; 19: 206.
